# Viral remodeling of the 4D nucleome

**DOI:** 10.1038/s12276-024-01207-0

**Published:** 2024-04-25

**Authors:** Kyoung-Dong Kim, Paul M. Lieberman

**Affiliations:** 1 Department of Systems Biotechnology, Chung-Ang University, Anseong, Korea; 2 The Wistar Institute, Philadelphia, PA 19104 USA

**Keywords:** Chromatin remodelling, Genetics research

## Abstract

The dynamic spatial organization of genomes across time, referred to as the four-dimensional nucleome (4DN), is a key component of gene regulation and biological fate. Viral infections can lead to a reconfiguration of viral and host genomes, impacting gene expression, replication, latency, and oncogenic transformation. This review provides a summary of recent research employing three-dimensional genomic methods such as Hi–C, 4C, ChIA-PET, and HiChIP in virology. We review how viruses induce changes in gene loop formation between regulatory elements, modify chromatin accessibility, and trigger shifts between A and B compartments in the host genome. We highlight the central role of cellular chromatin organizing factors, such as CTCF and cohesin, that reshape the 3D structure of both viral and cellular genomes. We consider how viral episomes, viral proteins, and viral integration sites can alter the host epigenome and how host cell type and conditions determine viral epigenomes. This review consolidates current knowledge of the diverse host-viral interactions that impact the 4DN.

## Viral infections reshape genomic landscapes: a 3D perspective

This research examines how viruses can change the 3D structure of host DNA, focusing on the role of CTCF and cohesin proteins in altering both viral and cellular 3D DNA structures. The scientists used various techniques, including chromosome conformation capture and next-generation sequencing, to study the 3D structure of both viral and cellular genomes. They discovered that viral infections can cause changes in gene loop formation, chromatin accessibility, and shifts in genome compartments. These changes can affect gene activity and various biological processes, potentially leading to diseases like cancer. The research concludes that understanding the spatial and temporal aspects of viral infection is vital for a full understanding of virus biology and the cellular 4D nucleome. This could lead to new treatment opportunities to fight virus infection, persistence, and disease development. “This summary was initially drafted using artificial intelligence, then revised and fact-checked by the author”.

## Introduction

The four-dimensional nucleome (4DN) helps to elucidate the spatial architecture of the cell nucleus in four dimensions, three-dimensional (3D) space and time, and the impact of alterations in these structures on human disease^1^. The 4DN consortium is an NIH Common Fund project that began in 2015. After completing its first phase in 2020, the consortium is currently in its second phase until 2025. In its first phase (2015–2020), the consortium focused on technologies for determining the 3D structure of chromosomes in time and space and the interplay between genome organization and gene expression^1,2^. In the second phase (2020–2025), several 4DN projects are focusing on chromatin dynamics at different time scales, including cellular differentiation, cell cycle stages, senescence, pathogenic processes, stress responses, and viral infections^2,3^.

Viral infections can lead to various changes in epigenomic reprogramming within host cells. Such infections often result in alterations in DNA methylation, histone modification, chromatin accessibility, and 3D organization of viral and host genomes and can influence gene expression, cellular functions, and various biological processes^4–6^. Notably, these alterations are closely linked to the progression of cancer. Approximately 17% of cancers worldwide are caused by viral infection, such as human papillomavirus (HPV)-related cervical cancer; hepatitis B virus (HBV) or hepatitis C virus (HCV)-linked liver cancer; and Epstein‒Barr virus (EBV)-related lymphoma, nasopharyngeal carcinoma, and gastric cancer. Kaposi’s sarcoma is also associated with Kaposi’s sarcoma-associated herpesvirus (KSHV)^7^.

The 3D organization of genomes in response to viral infection is an emerging focus of 4DN research^2,3^. Recent 4DN studies have revealed that viral infections induce reorganization in gene loop formation among regulatory elements, alter chromatin accessibility, and provoke shifts between A and B compartments in the genome. This review compiles recent research that has applied 3D genomic methodologies to virology, emphasizing the role of CTCF, cohesin, and other chromosome-organizing factors in reshaping both viral and cellular 3D genomes. In general, the impact of viral infection on the 3D organization of host genomes varies according to the specific virus, the stage of viral infection or latency, and the host cell type. The many ways in which viruses can impact the 4DN are just beginning to be defined at the molecular level. Consequently, we provide a review of recent advances in 3D structural analyses of viral–host interactions and their functional significance.

### Application of 3D genomic tools in the field of virology

Methods derived from chromosome conformation capture (3C) reveal chromosomal topological organization across various frontiers in virology. Early studies detected the proximity of two regulatory regions within viral genomes by utilizing 3C-PCR^5,8–11^. However, with the advancement of NGS technologies, contemporary methods such as Hi–C now enables the detection of entire genomic associations^12^, though in many viral genomic studies, additional methods are required to identify associations between the viral genome and host genomic loci. For this strategy, methods such as circular chromosome conformation capture (4C) and Capture Hi–C (CHi–C) are valuable because they magnify or enrich specific associations linked to viral genomes^13–15^. Additionally, techniques such as ChIA-PET and HiChIP can be employed to identify genomic associations mediated by viral or cellular proteins^16–18^. The application of 3D genomic methodologies has significantly impacted various aspects of virology, which can be categorized into three main interactions: viral–viral associations, viral–host associations, and host–host associations modulated by viral infection.

**Fig. 1:**
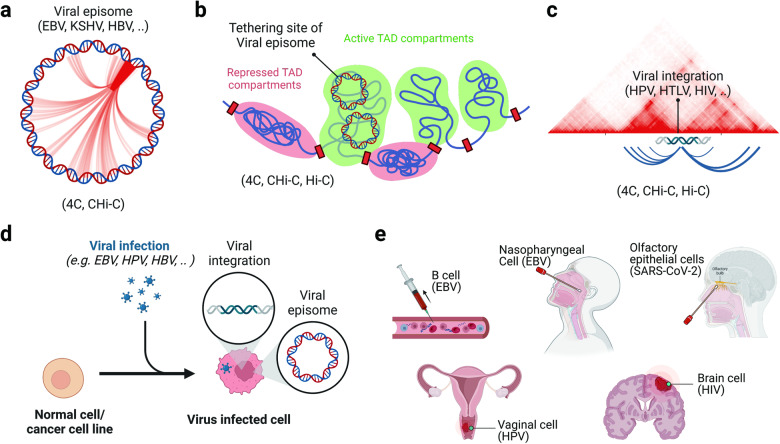
Application of 3D genomic methods in virology. **a** Overall, viral genomic associations with specific viral genomic regions can be captured by the 4C and CHi–C methods and are visually represented in a Circos plot. **b** Viral-host genomic associations are comprehensively captured by 4C, CHi–C, and Hi–C. Hi–C provides insights into the tethering site within the context of the 3D structure of the host genome. **c** Genomic associations between integrated viral genomes and host chromosomes were revealed by the 4C, CHi–C, and Hi–C methods. **d** Recent studies have induced viral infection in either cancer cell lines or primary cells in vitro, enabling further comparative 3D genomic studies. **e** Clinical samples obtained from patient biopsies, tissue banks, and autopsies have been utilized in recent studies to investigate 3D genomic aspects of viral infections.

Viral–viral genomic associations have been most extensively characterized in human DNA viruses, such as EBV and KSHV. These viruses persist as episomes in latently infected cells. Proper regulation of viral gene expression and stable maintenance of viral DNA during latent infection depends on chromatin structure and epigenetic programming^5,6,9^. In EBV and KSHV, CTCF and cohesin play pivotal roles in orchestrating the formation of loops between DNA regulator elements of viral genomes, significantly impacting transcriptional regulation^8,10,11^. To study the viral 3D genome, techniques such as 3C, 4C, CHi–C, and Hi–C have been utilized in numerous studies. Moreover, viral 3D genomes have been explored through genetic disruption of specific loci, such as CTCF binding sites, and in response to cell type variations and environmental factors^19–21^ (Fig. 1a).

Viral–host genomic associations have been best characterized using 3D genomic tools such as 4C, CHi–C, and Hi–C (Fig. 1b, c). Indeed, these methods have been instrumental in delineating the tethering sites of viral episomes to host chromosomes^20,22–24^. While 4C and CHi–C increase the resolution of associations with EBV episomes, Hi-C offers an understanding of these attachment sites within the context of the 3D structure of the host genome^18,22,23,25^. These tethering sites of viral episomes have been characterized in EBV, KSHV, and HBV, accounting for different cell types and latency patterns^4^. Tethering sites of viral episomes are often confined within distinct genomic structures, such as topologically associated domains (TADs)^23,26^ (Fig. 1b). Viral integration can also occur via interactions between viral and host 3D structures and regulatory elements.

Host‒host 3D genome interactions that change upon viral infection have been characterized for various virus infections and latency types. Remodeling of the host 3D genome was mapped by HiChIP and Hi–C for EBV-infected cells, and changes associated with B-cell immortalization, latency type, or disease status were compared^18,21,27–29^. Host 3D genome structures have also been characterized at the tethering sites of several episomal tumor viruses, including EBV, KSHV, and HBV, revealing cell type- and virus-specific variations^20,25,30,31^. In the context of integrating viruses such as human T-lymphotropic virus type 1 (HTLV), human immunodeficiency virus (HIV), and HPV, insertion of ectopic viral DNA into host chromosomes has been shown to alter cellular genomic loops and TAD structures^32–38^. Host 3D genome changes have also been observed for several viruses that do not interact with their host nuclear genome, such as SARS-CoV-2 and influenza A viruses, but modulate 3D genome changes through the action of viral proteins and other unidentified factors that are yet to be fully understood^39–41^ (Fig. 1c, d). Some of these studies have been carried out in established cell lines with static latent infections; others have examined changes over a time course of viral infection^18,22,23,26,28,30,31,40–44^ (Fig. 1d). Additionally, host 3D genomes were analyzed from clinical samples obtained from patient biopsies, tissue banks, and autopsies^32,37,45,46^ (Fig. 1e).

### The viral infection-induced 3D genome modulated by CTCF and cohesin

Cellular factors, such as CCCTC-binding factor (CTCF) and cohesin, have well-established roles in organizing chromatin within viral and host genomes^47,48^. CTCF and cohesin can also prevent the spread of repressive or active chromatin from one regulatory domain to another, effectively acting as chromatin insulators^9,49^. Furthermore, CTCF and cohesin play key roles in generating DNA loops, as exemplified by their involvement in facilitating interactions between enhancers and promoters^5,9^. CTCF and cohesin can also form boundaries between large topologically associating domains (TADs)^50–52^. In this section, we provide a brief overview of the role of CTCF and cohesin in the formation of both viral and host genome spatial architectures and their corresponding functions in regulating gene expression.

#### Role of CTCF and cohesin in regulating viral gene expression

**Fig. 2:**
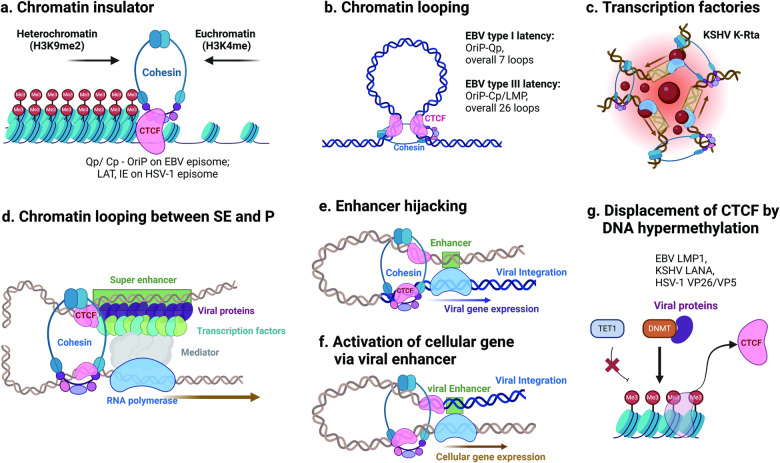
CTCF/cohesin-mediated 3D structure of the host and viral genomes. **a** CTCF and cohesin binding act as chromatin boundary elements, preventing the spread of euchromatin markers (H3K4me) as well as CpG DNA methylation and heterochromatin markers (H3K9me2) within EBV and HSV-1 episomes. **b** CTCF and cohesin orchestrate chromatin looping based on EBV latency type. **c** CTCF and cohesin establish multiple genomic loops, known as transcription factories, at sites where K-Rta binds within the KSHV episome. **d** CTCF and cohesin facilitate the formation of genomic loops between super-enhancers and the promoters of numerous genes. **e** Enhancers positioned in the viral genome can hijack host enhancers to express viral genes. **f** Enhancers positioned in the viral genome can activate cellular genes through the formation of loops between viral enhancers and host regulatory regions. **g** The EBV protein LMP1, KSHV protein LANA, and HSV-1 protein VP26/VP5 interact directly or indirectly with the host DNA methylation machinery, contributing to host DNA methylation and resulting in the dissociation of CTCF.

A chromatin boundary function of CTCF and cohesin in several viral genomes has been described. Occupancy of CTCF has been documented for double-stranded DNA viruses, such as EBV^8,53–55^, KSHV^11,56^, herpes simplex virus (HSV)^57,58^, HPV^59,60^, HTLV-1^35,36^, and human cytomegalovirus (HCMV)^61^, particularly during periods of latent infection, and modulates accessibility of the viral genome to transcription factors. In EBV episomes, CTCF binding upstream of the EBNA1 promoter, Qp, blocks the spread of CpG-DNA methylation and the heterochromatin marker H3K9me3 to prevent transcriptional silencing and loss of EBNA1 expression^5,8,62^. Additionally, CTCF binding sites positioned between the EBNA2 promoter Cp region and the OriP region can restrict the spread of H3K4me3 euchromatic histone modifications and activation of EBNA2 during latency type I^5,55,62^. In the case of KSHV episomes, both CTCF and cohesin bind to critical chromatin boundary elements upstream of the transcriptional regulatory elements of the lytic activator ORF50/K-Rta gene, which results in bivalent histone modifications^11^; moreover, within the first exon of the multicistronic latency gene, CTCF and cohesin control LANA and miRNA expression^56,63^. Consequently, the binding of CTCF and cohesin to chromatin boundary elements appears to be a general strategy employed by double-stranded DNA viruses to regulate viral genes in response to the status of viral latency (Fig. 2a).

CTCF and cohesin play pivotal roles in establishing chromatin loops that regulate gene expression. In EBV episomes, CTCF and cohesin mediate multiple DNA loops that regulate viral gene expression and latency. Early 3C studies revealed a prominent loop between OirP and LMP1p in type III latency, while in type I latency, OriP forms a more prominent loop with Qp. These loops correlate with differential promoter utilization in response to OriP enhancer function^9,10^ (Fig. 2b). More recent studies using CHi‒C have identified approximately 16 to 17 CTCF binding sites and numerous chromatin loops^18^. Isogenic type I and III EBV genomes exhibit nearly identical CTCF and cohesin binding profiles. Nevertheless, the 3D conformations of type I and type III genomes differ markedly, with only 7 DNA‒DNA interactions in type I compared to 26 in type III^21^ (Fig. 2b). In addition to CTCF binding sites at OriP, Qp, Cp, and LMP, a CTCF binding site located within the BamHI A right transcript (BART) miRNA promoter locus has been associated with overall EBV genomic loci and linked to expression of EBV BART miRNA and viral infectivity^19^.

In KSHV episomes, viral genomic loops are established by CTCF and cohesin complexes. Disruption of such genetic interactions by ablation of components of the cohesin complex deregulates the KSHV latency-lytic switch, leading to viral reactivation^64,65^. Cellular perturbations, such as ER stress-triggered Rad21 cleavage or the use of a BET inhibitor, can also alter the latent genomic conformation, and these alterations facilitate the transition to the lytic state by promoting RNA polymerase II interaction with particular loci^66,67^. Comprehensive analysis through CHi-C techniques revealed intricate intragenomic associations within the KSHV episome^68^. These associations are governed by the presence of CTCF, the cohesin complex, and the KSHV transactivator K-Rta. The switch to KSHV lytic replication is primarily initiated by the expression of a single viral protein, K-Rta. CHi‒C analysis has revealed the establishment of multiple genomic loops at sites where K-Rta binds, and induction of K-Rta expression amplifies the formation of these genomic loops, commonly referred to as transcription hubs or factories^68^ (Fig. 2c). As KSHV transitions from latent to lytic, its 3D structure undergoes reorganization to enhance recruitment of the K-Rta complex and activation of viral lytic cycle transcription^69^. Consequently, KSHV employs a spatiotemporal gene regulatory mechanism to ensure efficient viral gene expression.

CTCF binding motifs have also been identified in the genomes of HSV and CMV^61,70–72^. In the case of HSV-1, there are seven putative CTCF insulators located at both ends of the latency-associated transcript (LAT) enhancer and the immediate-early (IE) region. CTCF binding to CTRL2 within the LAT coding sequence is essential for maintaining chromatin at LAT and ICP0 regions, promoting efficient reactivation^70^. Deleting the CTRL2 insulator leads to a reduction in Rad21 enrichment, indicating that CTCF-cohesin interactions play a role in creating and anchoring chromatin loop structures to regulate viral transcription^71^. Additionally, the absence of the CTRL2 insulator resulted in HSV-1 establishing an equivalent latent infection in rabbits; however, these rabbits exhibited inefficient reactivation from latency^72^. Within the human cytomegalovirus (HCMV) genome, CTCF binds to the first intron of the major immediate-early (MIE) gene, playing a role in suppressing MIE gene expression. Removal of the CTCF binding site results in an increase in MIE gene expression and enhances viral replication. Furthermore, this CTCF binding motif is conserved in various CMVs, including murine CMV (MCMV)^61^.

#### Alterations in CTCF-cohesin loop formation in the host genome due to viral infection

CTCF and cohesin play crucial roles in establishing insulators at boundaries adjacent to TADs. Notably, in mammals, only 15% of CTCF-binding sites are situated at the borders of TADs; the remaining 85% are found within TADs^73^. Multiple studies have provided evidence that CTCF and cohesin within TADs direct the association between enhancers and promoters.

An earlier study revealed 187 EBV super-enhancers (ESEs) within EBV-transformed LCLs based on enrichment of EBV transcription factors (EBNAs) and participation of five EBV-activated NF-κB subunits, accompanied by distinct higher and broader histone H3K27ac signals^74^ (Fig. 2d). These 187 ESEs were associated with 544 genes, as explored through RNAPII ChIA-PET, with a significant representation of genes crucial for LCL growth and survival^75^. Notably, approximately 30% of the genes essential for LCL growth and survival were connected to an ESE via the formation of cohesin-mediated loops^75^ (Fig. 2d). These findings suggest that EBV has evolved a mechanism to selectively remodel 3D genome interactions to activate target genes that are pivotal for achieving immortal growth and survival.

CTCF binding sites have also been described among viruses that frequently integrate into the whole genome, such as HPV^32–34^ and HTLV^35,36^. Integration of HPV changes CTCF binding patterns and chromatin loops in the vicinity of integration sites^34^. Extensive CHi–C analysis has revealed that chromatin interactions between the integrated viral genome and host chromatin occur at both short (less than 500 kb) and long (greater than 500 kb distances), and areas where host chromatin interacts intensely correlate with host CTCF-binding sites. Furthermore, gene expression near HPV integration sites tends to become dysregulated^33^. Virus-encoded enhancer-like elements may activate nearby host genes via loop formation, and reciprocally, host enhancers may activate viral oncogenes through DNA loop formation (Fig. 2e, f). In the context of HTLV-1, a single CTCF binding site in the pX region of the provirus has been found to interact with flanking host chromatin. These CTCF insertions from the HTLV-1 genome result in pervasive disruptions to the chromatin structure of host T cells, leading to alterations in gene expression patterns and leukemagenesis^35,36^.

CTCF preferentially binds to unmethylated DNA^76,77^. In EBV-positive nasopharyngeal and gastric carcinomas, there is a high frequency of CpG island hypermethylation at various genomic loci, including tumor-suppressor genes^78–80^. Although the precise mechanisms governing DNA methylation by EBV are incompletely understood, EBV-encoded LMPs have been shown to induce host DNMTs, resulting in hypermethylation of both the host and viral genomes^78^. In addition, several viral proteins, including KSHV LANA^81^ and HSV-1 VP26/VP5^82^, interact with the host DNA methylation machinery and modulate host DNA methylation. This altered methylation landscape creates an unfavorable environment for CTCF binding and can alter the host 4DN (Fig. 2g).

### Restructure of the host 4DN by episomal DNA tumor viruses

#### Latency-type-specific reorganization of host chromatin status via viral infection

Episomal DNA tumors viruses, such as EBV, KSHV, and HPV, have the unique property of tethering to the host chromosome through their dedicated EMPs^4,25^. These EMPs have sequence-specific DNA-binding domains and separate chromosome tethering domains. The tethering domains of each of EMPs (EBNA1, LANA, and E2) are structurally distinct and have different affinities for host target proteins and nucleic acid structures^4,25^. The host 3D genome architecture may determine the site of viral episome tethering, and conversely, viral tethering may remodel the host 3D genome organization. EBV episomes tether diverse regions of host chromosomes that appear to differ according to latency state and cell type. EBNA1 is essential for episome tethering, and depletion of EBNA1 results in dissociation of EBV episomes from host chromosomes^4,20^. In EBV latency-type cells, such as MutuI and Raji cells, where EBNA1 is the only viral protein expressed, EBV episomes tether to repressed chromatin regions that are enriched by the histone marker H3K9me3 and AT-rich sequences^20^. Many of these host genome tethering sites also colocalize with sequence-specific recognition sites for EBNA1 and overlap with EBNA1 ChIP-seq binding sites. The EBNA1 tethering domain contains an AT-hook structure that is likely to facilitate tethering to AT-rich DNA^20^. Intriguingly, H3K9me3 enrichment decreases when EBNA1 is depleted, suggesting that tethering of EBV episomes may reinforce the repressive and compact heterochromatin status^20^.

**Fig. 3:**
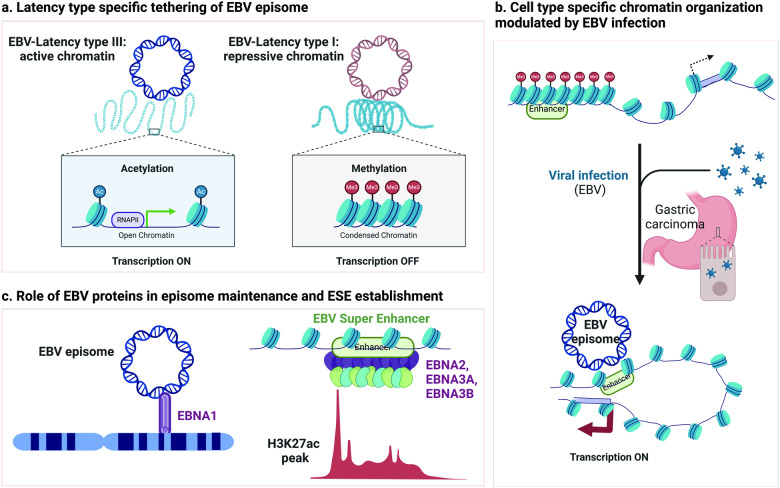
Topological relationship between the EBV episome and host chromosome. **a** During latency type III, viral chromatins exhibit activity, and the EBV episome is associated with active open chromatin regions. In contrast, during latency type I, viral chromatin is repressed, and the EBV episome is linked to repressive heterochromatin regions. **b** In EBV-associated gastric cancer (EBVaGC), tethering of the EBV episome results in the conversion of heterochromatin to active euchromatin, leading to the activation of dormant enhancers. **c** The EBV protein EBNA1 tethers the EBV episome to specific loci on the host chromosome. EBV transcription factors, such as EBNA2, EBNA3A, and EBNA3C, collaborate with host factors to establish the EBV super-enhancer (ESE) on host chromosomes.

The location of EBV episome tethering sites appears to be dependent on the latency type (Fig. 3a). In lymphoblastoid cell lines (LCLs) with latency type III, EBV episomes were found to tether to active chromatin, which is marked by H3K27ac and H3K4me1/3^20,25^. Although the impact of EBV episomes on the tethering of active chromatin has not been characterized, the global genome reorganization induced by EBV infection has been studied^18^. Primary human resting B lymphocytes (RBLs) can be transformed into immortal LCLs by infection with EBV^83^. Comparative analyses using Hi-C data from RBLs and LCLs have revealed that EBV infection triggers the restructuring of the host chromosome configuration^18^. In detail, the inactive B compartment in RBLs often switches to the active A compartment in LCLs. Moreover, LCLs show the formation of new contact domain boundaries, indicating increased local interactions in comparison to RBLs, particularly at LCL dependency factors and super-enhancer targets^18^. It is not yet known whether viral genome tethering directly contributes to such host genome 3D remodeling. Nevertheless, these studies suggest that viral tethering sites vary depending on cell type and that the chromatin environment correlates with euchromatic environments for more permissive type III latency and with heterochromatic environments for more restrictive type I latency.

Episome-binding sites have been described and partly characterized in several other viruses. KSHV episome tethering to host cell chromosomes is maintained by the KSHV protein LANA^4^. The N-terminus of LANA interacts with host chromosomes through interactions with histones H2A and H2B^84^. Additionally, the DNA-binding domain of LANA interacts with host chromatin-associated proteins, including BRD2 and BRD4^85^. CHi–C analysis has revealed that the tethering sites of KSHV episomes are notably enriched near centromeric regions^24^. This observation is consistent with a previous report indicating that LANA colocalizes with centromeric protein F (CENPF) and kinetochore protein (Bub1)^86^. Furthermore, KSHV episomes tend to associate with the cellular proteins CHD4 and ADNP at sites enriched with H3K27ac^24^. For HPV, the viral protein E2 binds to ribosomal DNA and the pericentromeric region of host chromosomes and recruits HPV episomes to these specific loci^87,88^. The tethering sites of HBV cccDNA are linked to transcriptional regulation of the viral genome^26,30,42^. Transcriptionally active HBV cccDNA is associated with transcriptionally active host chromatin, while transcriptionally inactive HBV cccDNA tends to localize preferentially to the five heterochromatic regions of human chromosome 19^30^.

#### Cell type-specific genome organization via viral infection

In B lymphoid cells, EBV infection leads to the rewiring of genomic loops between the ESE gene and regulatory regions of genes, including MYC (a proto-oncogene)^89^, BCL2L11 (encoding the pro-apoptotic Bcl-2 family binding protein Bim)^89^, AICDA (encoding the B-cell protein AID regulating class switch recombination and somatic hypermutation)^18^, and genes associated with leukocyte cell‒cell adhesion (NAALADL2-AS2, CD80, SLAMF1, and ZMIZ1)^28^. This rewiring is a crucial factor in driving lymphoma development.

EBV is implicated in the development of two major epithelial malignancies, gastric carcinoma and nasopharyngeal carcinoma^22,23,45^. Studies focusing on EBV-associated epithelial malignancies have revealed that EBV episomes target specific genomic regions distinct from those found in lymphoid cell lines^23^. In gastric carcinoma cells, EBV infection induces the reorganization of genomic loops between ESE and the regulatory region of genes such as TGFBR2 (associated with epithelial to mesenchymal transition) and MZT1 (mitotic spindle organizing protein 1)^23^. In addition, EBV infection in gastric carcinoma cells leads to repression of LINE-1, resulting in genome instability and, in some cases, disease^22^. The tethering sites of EBV episomes in EBV gastric cancer are significantly associated with an increase in compartments undergoing a B-to-A transition. This finding suggests that EBV episomes interact with the inactive B compartment and, in specific regions, modify their interaction areas toward the active A compartment^23^. Consistent with these findings, tethering of the EBV episome leads to the conversion of H3K9me3 heterochromatin to H3K4me1/H3K27ac bivalency, consequently activating dormant enhancers (Fig. 3b). This action enables these enhancers to activate nearby genes, such as KLF5 and TGFBR2, related to gastric cancer^23^. KLF5 has been shown to facilitate GC development^90^, and TGFBR2 has been shown to be involved in regulating the epithelial-to-mesenchymal transition^91^.

In EBV-infected MKN (a GC cell line), the EBV episome distinctly binds near the transcription activation suppressor (TASOR) enhancer, which enhances loop formation between the TASOR promoter and enhancer. This leads to the upregulation of TASOR. Upregulated TASOR expression facilitates assembly of the human silencing hub (HUSH) complex, which further promotes the deposition of H3K9me3 on LINE-1, effectively silencing its expression^22^. Dysregulation of LINE-1-mediated retrotranspositions is closely linked to diseases, including autoimmune disorders, neural diseases, and cancers^22^.

In many EBV-positive nasopharyngeal carcinomas (NPCs), EBV establishes global DNA hypermethylation, leading to a decrease in CTCF binding and an alteration in chromatin accessibility. However, some enhancers, CpG islands (CGIs), and promoters exhibit increased chromatin accessibility, suggesting a high level of epigenetic heterogeneity across NPC tumors^45^. RNA-seq and ATAC-seq data show that CD74 is significantly upregulated in NPC tumors with a hypomethylation phenotype, and this change correlates with markers of T-cell exhaustion and immune evasion^45^. Furthermore, EBV infection in nasopharyngeal cells results in increased chromatin accessibility at the promoter of CD74, which is involved in T-cell exhaustion^45^. However, whether these epigenetic features are related to episome tethering is not yet known.

Other viruses with different tethering mechanisms also modulate host chromatin and gene expression. KSHV infection of PEL cells triggers activation of disease identity genes, including CCND2, MYC, MYB, and PIK3C3, and PSOD genes, which are mediated by super-enhancers^92^. It is not yet known whether KSHV tethering through LANA is directly related to host 4DN remodeling. HBV cccDNA preferentially links to CGIs, which are associated with highly expressed genes and genes deregulated during infection, particularly those related to liver disease^31^. Hepatoblastoma cell lines infected with HBV undergo a reorganization of the host 4DN, resulting in dysregulation of metabolic liver-associated pathway genes and downregulation of tumor suppressor genes such as PROS1 and NLGN4Y^93^. Thus, tethering of the viral episome and its influence on host chromatin and gene expression vary largely depending on the host cell type.

### Viral proteins related to the host 4DN

Many virus-encoded proteins are known to modulate host chromatin and influence 3D genome structure. Viral-encoded EMPs, such as EBNA1 of EBV, LANA of KSHV, and E2 of HPV, have bivalent DNA-binding properties and can therefore create DNA loops and bridges between genomes. EBNA1 contains specific domains, a carboxy-terminal DNA-binding domain, and an amino-terminal RGG/AT-hook domain, allowing it to tether viral episomes and host chromosomes, respectively^4^ (Fig. 3c). LANA and E2 exhibit structural characteristics similar to those of EBNA1 and bind to both viral and host genomes^4,25^. In addition to EMPs, viral proteins can impact the host chromatin environment. The EBV transcription factors EBNA2, EBNA3A, and EBNA3C establish ESEs with host factors^18,28^ (Fig. 3c). HiChIP with H3K27ac in LCLs has revealed extensive long-range looping interactions between ESEs and the regulatory regions of host genes, including oncogenes. The roles of the EBV transcription factor EBNA2 and EBNA3A/C in the organization of the host genome have been investigated by conditionally expressing or depleting viral proteins. Through ATAC-seq and HiChIP with H3K27ac, more than 2,000 regions of EBNA2-dependent chromatin accessibility and more than 1700 regions of EBNA2-mediated chromatin looping interactions have been identified, resulting in the expression of more than 400 human genes dependent on EBNA2, including autoimmune genetic risk loci such as ZMIZ1^28^. Another HiChIP study revealed a significant decrease in the number of genomic loops containing enhancers, promoters, and CTCF binding sites in LCLs when EBNA3A was inactivated. Consequently, the inactivation of EBNA3C disrupts local CTCF interactions at the CDKN2A/B and AICDA loci, subsequently elevating the expression of these genes^18^. Similarly, in KSHV-transformed primary effusion lymphoma (PEL), viral interferon regulatory factor 3 (vIRF3) of KSHV, along with the cellular factors IRF4 and BATF, co-occupies the super-enhancer of critical survival genes, driving upregulation of PEL-specific cellular oncogenic dependency (PSOD) genes through super-enhancer mediation^92^.

RNA viruses can also interact with host chromatin to modulate viral replication. For example, the influenza A protein NP interacts with the histone methyltransferase Suv4‒20h2, resulting in the inactivation of Suv4‒20h2 and subsequent dissociation of cohesin. This, in turn, allows cohesin to mediate the active loop formation of HoxC8‒HoxC6, leading to increased expression of HoxC8 and HoxC6^41^. In response, the HoxC8 and HoxC6 proteins boost viral replication by inhibiting the Wnt-β-catenin-mediated interferon response^94,95^.

### Restructuring of the host 4DN by integration of the viral genome

Furthermore, viral integration can have a significant impact on the host 4DN. Integration is known to cause chromosomal instability and induce gene rearrangement and copy number variation, which can be pathogenic^96,97^. Recent studies have illustrated the response of the host 3D genome landscape to viral integration, particularly for HPV, HTLV, and HIV.

**Fig. 4:**
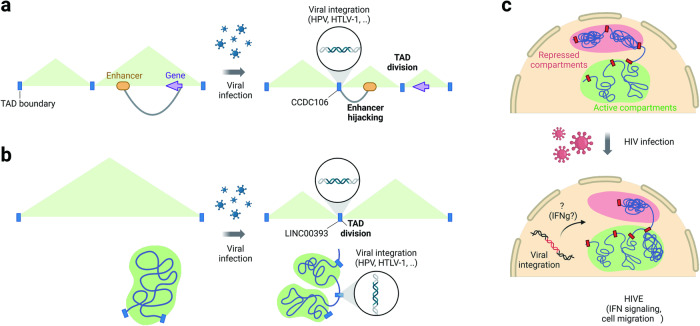
Viral integration-induced restructuring of the host 4D nucleome. **a** HPV infection triggers the integration of the viral genome into integration hotspots, as exemplified by CCDC106. Integration at CCDC106 preferentially associates with the enhancer of the PEG3 gene, leading to the division of TADs. **b** HPV integration within TADs can lead to the splitting of these domains. **c** Analysis of Hi‒C data from tissue samples of individuals with HIV and encephalitis revealed a shift in compartment structure from B to A, and this shift correlated with the derepression of genes associated with IFN signaling and cell migration pathways. Comparative changes in compartment structure were observed in microglia stimulated with IFNγ. These findings highlight the impact of viral integration on the 3D genome architecture and its implications for gene expression and cellular pathways.

Progression of cervical carcinoma is strongly linked to the integration of the HPV genome into host chromosomes, leading to dysregulation of HPV oncogene expression^32–34^. HPV tends to integrate into specific genomic sites known as integration hotspots, such as the CCDC106 gene chromosome 19 and LINC00393 on chromosome 13^32,34^. Through comparative Hi–C analysis, it was observed that HPV integration at the CCDC106 site divides a TAD into two smaller TADs within carcinoma cells^32^. This division leads to a significant reduction in the interaction of the enhancer with the PEG3 gene promoter and an increase in the interaction with the CCDC106 gene promoter (Fig. 4a). Consequently, this downregulates PEG3 expression and upregulates CCDC106 expression^32^. PEG3 is known for its tumor-suppressor activity in various cancer types and contributes to p53 activation^98–100^. HPV integration at the LINC00393 site also induces TAD division, subsequently resulting in the downregulation of KLF12, a well-known tumor-suppressor gene^34^ (Fig. 4b). Additionally, dysregulation of the ARL15 and ITGA2 genes, both of which are linked to tumorigenesis, occurs in the vicinity of the HPV integration site^33^. Hence, dysregulation of genes related to tumors due to alterations in genomic loops near HPV integration hotspots might play a role in the development of cervical carcinogenesis.

Integration of human T-lymphotropic virus type 1 (HTLV-1) into host chromosomes disrupts host chromatin structure by establishing loops between the provirus and the host genome, as facilitated by CTCF-mediated enhancer hijacking^35,36^. Given that CTCF binds to numerous loci across the human genome, the introduction of a CTCF-binding site by HTLV-1 has the potential to induce broad abnormalities in the chromatin structure and gene expression of host cells^36^.

In contrast, HIV does not contain a CTCF binding site; integration occurs more frequently at CTCF-enriched TADS, and depletion of CTCF reduces integration efficiency^101,102^. The state of the host chromatin and location of integration are crucial factors influencing HIV-1 gene regulation^37,38^. Analysis of Hi–C data obtained from tissue samples of individuals with HIV encephalitis (HIVE) revealed a notable association between transcriptional derepression of interferon signaling and cell migratory pathways, as did a shift in A/B compartment structures from B to A^37^. In a specific set of locations, comparative changes were observed in cultured microglia stimulated with IFNγ, indicating that IFN stimulation contributes, at least in part, to the 3D remodeling observed in HIVE^37^ (Fig. 4c). Another investigation using latently HIV-1-infected Jurkat T cells demonstrated that activation of HIV-1 transcription leads to heightened chromatin accessibility downstream of the HIV-1 genome, extending it by 1–10 kb^37^. Overall, the impact of HIV integration on the local or global reorganization of chromatin structure appears to depend on cell type and transcriptional activation.

### Additional mechanisms of 3D genomic alterations caused by viral infections

Human adenoviruses replicate productively in the nucleus of many different cell types and can remodel nuclear chromatin at the microscopic level, suggesting a reorganization of the host 4DN^103^. Hi-C and CHi–C were used to infect primary human hepatocytes (PHHs) infected with adenovirus type 5 (Ad5) to identify the contact sites of adenoviruses across host chromosomes and the effect of viral attachment on the expression of nearby genes^31^. This approach is crucial because adenoviruses are extensively utilized as viral vectors to deliver desired genes to the nucleus of target cells. Notably, Ad5 DNA tends to make contacts primarily with transcription start sites (TSSs) and enhancers associated with highly expressed genes^31^. During infection, genes associated with Ad5 tend to be upregulated. Furthermore, the Ad5 virus induces dynamic changes in the host 4DN, leading to the reshaping of host TADs and a shift in the A/B compartment from B to A. Notably, Ad5 DNA attachment sites are associated with motifs for FOXA1, FOXA2, and CAAT enhancer-binding protein (C/EBP), and FOXA proteins recruit mediators and cohesin^104^. Hence, exploring the 3D genome structure mediated by cohesin and mediators after Ad5 viral infection would be an intriguing avenue for research.

HBV can modulate host chromatin through viral–host protein interactions. The HBV-encoded HBx protein can promote degradation of the host structural maintenance chromosome protein (SMC)5/6 complex, which functions as a host restriction factor^105^. Intriguingly, HBx may antagonize the host SMC5/6 complex to facilitate the localization of HBV cccDNA near active chromatin regions. Thus, positioning of the HBV episome is regulated by both HBx and the SMC5/6 complex^30,42,106^.

White spot syndrome virus (WSSV) is a marine dsDNA virus that has been shown to modulate the host 4DN structure through protein mimicry^107^. The WSSV-encoded VP9 protein forms dimers that mimic DNA and hinder the binding of histones to DNA. When VP9 is expressed in HeLa cells, increased mobility and solubility of histones result, leading to relaxed compaction of the chromatin structure. Moreover, VP9 expression induces significant alterations in host gene expression^107^. Nonetheless, the detailed mechanism underlying VP9-mediated chromatin decompaction and the ultimate viral strategy have not been fully elucidated.

RNA viruses, such as influenza A (IAV), can also induce significant reshaping of the host 4DN. The IAV NS1 protein hinders the termination of transcription, resulting in the production of extended readthrough transcripts. These readthrough transcripts have significant effects, including local chromatin decompaction and frequent transition from B to A compartments, particularly downstream of highly transcribed genes. Mechanistically, these readthrough transcripts displace cohesin from chromatin, altering the 3D genome structure by disrupting cohesin-mediated loops following IAV infection^44^. Interestingly, a similar phenomenon of readthrough transcription was observed in HSV infection^108^. In HSV-1 infection, the ICP27 protein obstructs host gene transcription termination by inhibiting the 3’ processing factor CPSF. This action is subsequently followed by ICP22 protein-dependent loss of histones downstream of the affected genes^108^. It would be intriguing to investigate the position of cohesin and the 4DN structure in response to HSV-1 infection compared to the IAV scenario.

Recent research findings indicate that SARS-CoV-2 infection disrupts epigenetic regulation and leads to restructuring of the host 3D genome organization^39,40,46^. The SARS-CoV-2 protein ORF8 functions as a histone mimic of the ARKS motif in histone H3 that disrupts host cell epigenetic regulation and promotes chromatin compaction^39^. SARS-CoV-2 infection triggers rearrangement in the chromosome architecture of olfactory sensory neurons, concurrently resulting in the downregulation of genes associated with odor perception. This discovery illuminates the molecular basis of SARS-CoV-2-induced anosmia^46^. In another study using human lung adenocarcinoma A549 cells infected with SARS-CoV-2, viral infection notably altered the host chromatin3D structure^40^. This alteration involves widespread weakening of compartment A, mixing of A and B compartments, reduced intra-TAD contacts, and decreased H3K27ac levels^40^. Changes in 3D genome structures are linked to transcriptional suppression of interferon response genes, and an increase in H3K4me3 is observed in the promoters of proinflammatory genes^40^. Furthermore, SARS-CoV-2 weakens the loading of cohesin in intra-TAD regions. This weakening may be linked to a reduction in intra-TAD contraction and an increase in long-distance chromatin interactions^40^.

## Conclusion

Viral infections perturb many host processes, but ultimately, there is a conflict between two genomes with different structural properties and reproductive strategies. How viruses perturb the 3D genome of the host and how 3D genome dynamics regulate viral infection remain important areas of investigation. New methods have been developed to better assess chromatin architecture and spatial genomic organization at higher resolutions and to correlate this information with functional readings for each genome. Viral and host genomes interact at many levels, including via episomal tethering and genome integration, which create new local chromatin environments that influence different viral and host genes. Many virus-encoded proteins can modulate host and viral 3D genomes through diverse mechanisms, including direct DNA binding and indirect mechanisms, such as DNA mimicry and modification of cellular structures and enzymes. All these perturbations to the virus‒host 3D genome have implications for gene expression, replication, and genome stability. Thus, investigating the spatial and temporal dimensions of viral infection is critical for obtaining a complete understanding of viral biology and the cellular 4DN.

There are many unanswered questions, and our knowledge of how CTCF and cohesin DNA loop interactions work in conjunction with chromatin remodeling and DNA methylation patterning in viral and host genomes is incomplete. A better understanding of the impact of episome tethering and viral genome integration on host chromosome epigenetic patterning, including histone modifications, 3D genome reorganization, and TAD formation, is also critical for understanding viral–host interactions. Overall, how 4DN interactions regulate the cellular response to viral infection, including intrinsic resistance and the interferon response, requires further clarification. Understanding the cell type-specific differences in the 4DN and how these differences impact viral infection and gene regulation is also essential for understanding viral tropism and cell type permissivity, including latent-lytic pathway decisions. How viral proteins and noncoding RNAs impact the host 4DN has not been fully elucidated and may provide insight into viral pathogenesis and new opportunities to manipulate cells for gene therapy or oncolytic strategies. Consequently, future analyses incorporating comprehensive 4DN data may provide valuable insights into the potential mechanisms that drive virus-mediated disease and provide new therapeutic opportunities to combat virus infection, persistence, and pathogenesis.
